# Thinking about others and the future: Neural correlates of perspective taking relate to preferences for delayed rewards

**DOI:** 10.3758/s13415-017-0550-8

**Published:** 2017-11-13

**Authors:** Garret O’Connell, Chun-Ting Hsu, Anastasia Christakou, Bhismadev Chakrabarti

**Affiliations:** 1 Centre for Integrative Neuroimaging and Neurodynamics, School of Psychology and Clinical Language Sciences, University of Reading, Reading, RG6 6AL UK; 2 Berlin School of Mind and Brain, Humboldt-Universität zu Berlin, Berlin, Germany; 3 Brain, Language, and Computation Lab, Department of Psychology, Pennsylvania State University, State College, PA USA

**Keywords:** Intertemporal choice, Perspective taking, False belief, Egocentric bias, Temporal discounting, Temporoparietal junction

## Abstract

We infer the thoughts and feelings of others by taking their perspectives. Similar processes could be used to understand how we will be affected by future events, by allowing us to take the perspective of our future self. In this paper, we test this idea using a previously presented framework for guiding predictions. The framework proposes that a shared neural mechanism is involved in controlling egocentric bias, both while shifting our perspective away from self and towards others, and while shifting our perspective from immediate to future perspectives. To test this framework, 36 adults performed an intertemporal choice task. They were then scanned using 3T functional magnetic resonance imaging while completing a false-belief “localizer” task, which requires egocentric bias control. A positive correlation was observed between the right temporoparietal junction (rTPJ) response during the false-belief task, and preferences for delayed rewards in intertemporal choices. A subset of participants performed the intertemporal choice task again in the scanner, which revealed that the response of the same rTPJ cluster, individually localized during the false-belief task, was higher during delayed over immediate reward choices. In addition, functional connectivity between the rTPJ and ventromedial prefrontal cortex was found to differ between immediate and delayed choices. The current results indicate an overlap in processes of egocentric bias control and those that determine preferences in intertemporal choices, offering a social cognitive explanation for why rewards are devalued with delay in temporal discounting.

**Electronic supplementary material:**

The online version of this article (10.3758/s13415-017-0550-8) contains supplementary material, which is available to authorized users.

The ability to see the world from different perspectives is useful in the context of our complex social environment. It allows us to take the perspectives of others and understand them. It may also be useful in an intertemporal context; for instance, when faced with decisions with delayed consequences, it could allow us to shift our perspective into the future to assess how these might impact us later. The relationship between our capacity for taking the perspective of others and those of our future selves has previously been speculated (Buckner & Carroll, 2007; Ersner-Hershfield, Wimmer, & Knutson, 2009; Jamison & Wegener, 2010; Mitchell, 2009), but how these processes are precisely related is not clear. To investigate this, we previously laid out a framework for how these capacities might relate to each other (O’Connell, Christakou, & Chakrabarti, 2015), called the simulation model of intertemporal preferences (SMIP).

The SMIP attempts to relate a marker of predicting future personal states in temporal discounting, to the ability to infer the minds of others in perspective taking. Temporal discounting describes the decrement in our preference for larger rewards as a function of the delay to their attainment. The rate of this devaluation of rewards with increasing delay is indexed by the steepness of the “discounting curve” (Ainslie, 1975). The economist George Loewenstein proposed that temporal discounting happens because it is more difficult to “empathize” with the feelings of enjoyment or expected benefit of rewards for more distant future selves (Loewenstein, 1996). The SMIP framework was built on this notion that social capabilities underpin the use of imagined future subjective states in reward-related decision-making.

Perspective taking is the ability to infer the thoughts, feelings, and beliefs of others. It can be compromised when we falsely presume that other people think or feel the same as us, a phenomenon termed “egocentric bias.” The ability to control this bias is commonly measured using “false-belief” tasks, in which people are required to make inferences about the beliefs of others, avoiding the tendency to erroneously assume others have access to the same information as them. Numerous experiments have studied false-belief tasks using functional Magnetic Resonance Imaging (fMRI), and these have highlighted the right temporoparietal junction (rTPJ) as a key brain region involved in the processing of these tasks (Krall et al., 2015). Further findings have indicated that better control of egocentric bias during false-belief tasks elicits stronger activity in the rTPJ (Dodell-Feder, Tully, Lincoln, & Hooker, 2013; Gweon, Dodell-Feder, Bedny, & Saxe, 2012; Kana, Keller, Cherkassky, Minshew, & Just, 2009), suggesting the potential of this response as a neural marker of egocentric bias control.

The SMIP hypothesizes that intertemporal choices are analogous to social situations in which egocentric bias occurs, in that there is an egocentric immediate perspective and a target future perspective to be constructed. From one’s immediate perspective, delayed rewards need to be waited for to be received, and therefore incur the cost of waiting, leading them to be represented as less pleasurable or beneficial than they actually would be felt in the future. Egocentric bias therefore contributes to this representational asymmetry by anchoring subjective evaluations of intertemporal choice options in the immediate perspective, from which delayed rewards incur a cost of waiting. Overcoming this egocentric bias when taking future perspectives therefore reduces this cost and increases preferences for delayed rewards in intertemporal choices.

The SMIP further proposes two interacting neural nodes in this mechanism of intertemporal egocentric bias control: (1) the rTPJ for the control of egocentric bias, based on the evidence outlined above, and (2) the ventromedial prefrontal cortex (vmPFC) in representing the expected pleasure or benefit of future rewards. This proposed function of the vmPFC is derived from empirical reports that demonstrate the role of this region in coding the subjective value of rewards, including delayed rewards (Frost & McNaughton, 2017). The vmPFC is also sensitive to the discounting effect of delay on subjective reward value (Jimura, Chushak, & Braver, 2013). Damage to this region causes reductions in both future perspective taking (Bertossi & Ciaramelli, 2016; Bertossi, Tesini, Cappelli, & Ciaramelli, 2016) and preferences for delayed over immediate rewards (Peters & D’Esposito, 2016; Sellitto, Ciaramelli, & di Pellegrino, 2010). Perhaps most directly relevant to the SMIP, a number of studies have now used a version of the temporal discounting task that cues participants to actively engage in future perspective taking during intertemporal choices. In comparison to the standard temporal discounting task, these studies report increased activation of the vmPFC and preferences for delayed rewards (Benoit, Gilbert, & Burgess, 2011; Hu, Kleinschmidt, et al., 2017; Hu, Uhle, et al., 2017; Peters & Büchel, 2010; Sasse, Peters, & Brassen, 2017; Sasse, Peters, Büchel, & Brassen, 2015).

Since the SMIP proposes that abilities of egocentric bias control interact with representations of future reward states, it implies that intertemporal preferences are influenced by coordinated signaling between the neural nodes it ascribes these functions to, i.e. the rTPJ and vmPFC respectively. However, it is unclear how such coordination might translate into choice behaviour. One possibility is that higher levels of this coordination enhance the value representations of future rewards, thereby promoting delayed reward choices. Another alternative is that such coordination indexes the effort to control egocentric bias, when posed with tempting immediate reward choices (i.e. through imagining the expected benefit of the reward).

In two experiments from Soutschek, Ruff, Strombach, Kalenscher, and Tobler (2016), repetitive transcranial magnetic stimulation (TMS) was used to disrupt participants’ rTPJ functioning. It was found that both the degree of egocentric bias in a visual perspective-taking task, and preferences for immediate rewards in a temporal discounting task, were subsequently increased. Furthermore, a positive relationship between egocentric bias and immediate reward preferences was observed across individuals. These effects indicate the involvement of the rTPJ in both egocentric bias control and in promoting preferences for delayed rewards, in line with the SMIP framework.

The current study aims to test key predictions of the SMIP and build on the findings of Soutschek et al. (2016) by examining the spatial overlap between neural processes of egocentric bias control in the rTPJ and temporal discounting, this time in terms of brain function using fMRI. If rTPJ response during perspective taking is a marker of egocentric bias control, as assumed by the SMIP and indicated by empirical work, people higher in this engagement should prefer delayed rewards more. This was tested using a false-belief functional localizer task, designed to identify the individually specific rTPJ cluster involved in egocentric bias control. In each individual, activity in this region during egocentric bias control was related to temporal discounting rates in an intertemporal choice task, done outside the scanner. Activity in this localized rTPJ cluster was also measured during intertemporal choices made in the scanner, to test if neural processes of egocentric bias control spatially overlap with those related to preferences for delayed rewards. Lastly, the SMIP claims that egocentric bias control in the rTPJ influences preferences in intertemporal choices by modulating representations of future reward value in the vmPFC. To test this neural mechanism, functional connectivity between the localized rTPJ cluster and the vmPFC was compared when immediate and delayed rewards were chosen.

## Materials and methods

### Participants

Thirty-eight adults (21 female, age range 18–34 years, mean age 22.6 years) were recruited and compensated £15. Participants gave informed consent, and the study was approved by the University Research Ethics Committee. Participants performed the tasks in the following order.

### Temporal discounting task (outside scanner)

In the first temporal discounting task outside the scanner, participants were informed that rewards were hypothetical and were instructed to not factor in their current financial situation during decision-making. Participants choose at a self-paced rate (1-s ITIs) between a variable amount of money now (<£100), or £100 at one of six randomly selected delays (months: 1, 3, 6, 9, 12, 18). Indifference points were calculated using the double-limits algorithm (Johnson & Bickel, 2002), and temporal discounting rates estimated as the area under the curve (AuC; see Supplementary Materials).

### False-belief localizer task (in scanner)

**Fig. 1:**
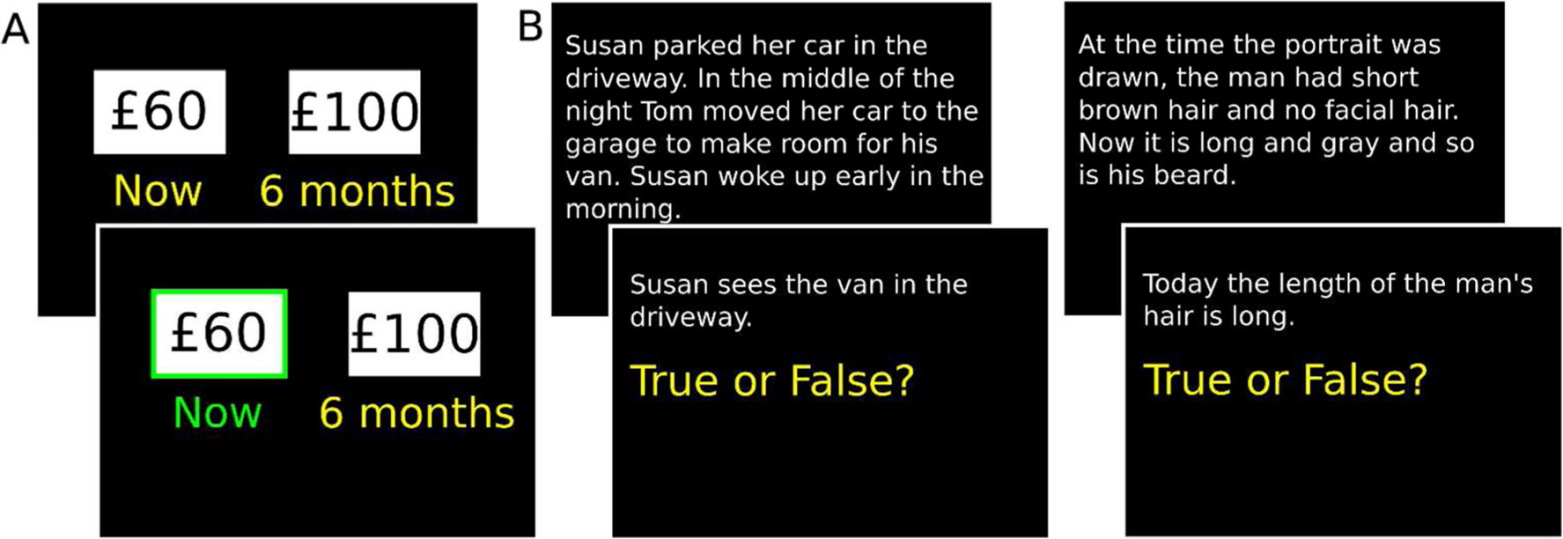
Example trials of (**a**) scanner temporal discounting task. **b** False-belief localizer task (left: false belief condition, right: FACT condition). (Color figure online)

The false-belief localizer task (http://saxelab.mit.edu/superloc.php; Dodell-Feder, Koster-Hale, Bedny, & Saxe, 2011) consisted of 10 short stories each about other people’s beliefs (false belief) and about historical facts (FACT, referred to as “photo” trials in the original task). Each trial started with a blank screen for 12 s, followed by the story for 10 s, and then a question screen for 4 s, which required participants to give a true or false response (see Fig. 1b).

#### fMRI data acquisition and analysis parameters (false-belief localizer task)

Scanning was conducted using a Siemens 3T Trio MRI scanner with an EPI sequence of TR 2 s, TE 30 ms, 2-mm^3^ voxels, and 37 interleaved 3-mm slices. Using FSL, data were field map unwarped, prewhitened, motion corrected, slice-time corrected, and high-pass filtered at 128 Hz and smoothed at 8-mm FWHM in native space. False belief and FACT trials were defined as the 14 s of the story and question screens (see Fig. 1b).

#### rTPJ false-belief localization procedure

An iterative threshold-adjusting procedure was adapted from (Dodell-Feder, DeLisi, and Hooker 2014) and Mitchell (2008) to localize individual rTPJ clusters. This involved increasing the height activation threshold of the False Belief > FACT contrast in native space in steps of 10^-1^, starting from *p* < .01 (cluster threshold *p* = .05) until a cluster in the rTPJ region was identified of 25–50 voxels in size. From these clusters, the percentage signal change in the False Belief > FACT contrast was extracted in native space (an estimate hereafter referred to as rTPJ_FB_) using Featquery in FSL. For thoroughness of reporting, this procedure was applied to other regions consistently activated by the false-belief task in the left temporoparietal junction (lTPJ) and precuneus.

#### Temporal discounting task (in scanner)

In the scanner, participants were presented with intertemporal choices featuring three delays (months: 6, 9, 12). Amounts of immediate options were presented in the value ranges £5 to £15 above and below the indifference points from the outside scanner data (see Supplementary Materials). This was done (a) to reliably predict choices so the number of trials (32) could be efficiently balanced between the two conditions in which immediate (IMM) or delayed (DEL) rewards were chosen, and (b) to ensure immediate options were close to indifference points, and therefore relatively difficult in terms of deciding preference, which we theorized engages perspective-taking abilities more than in trials where choices are easy (O’Connell et al., 2015). Options were presented together for 5 s, the selected option turned green (see Fig. 1a), and a jittered ITI of 7 s to 15 s followed. The task was run on PsychToolbox on MATLAB 2012b.

#### fMRI temporal discounting data acquisition and analysis

Twenty-six out of the total 38 participants performed the scanner version of the temporal discounting task. Scanning was conducted with an EPI sequence of TR 3 s, echo time 30 ms, 2 × 2 × 2-mm^3^ voxel size, and 35 interleaved 3-mm slices. Data were preprocessed using SPM8, with slice-timing correction, realignment for motion correction, field map unwarping, and sequential coregistration. Structural images were tissue segmented and used to create a group template with DARTEL toolbox. Transformation parameters for structural images were applied to normalize functional images to MNI space, and smoothed at 6-mm FWHM.

**Fig. 2:**
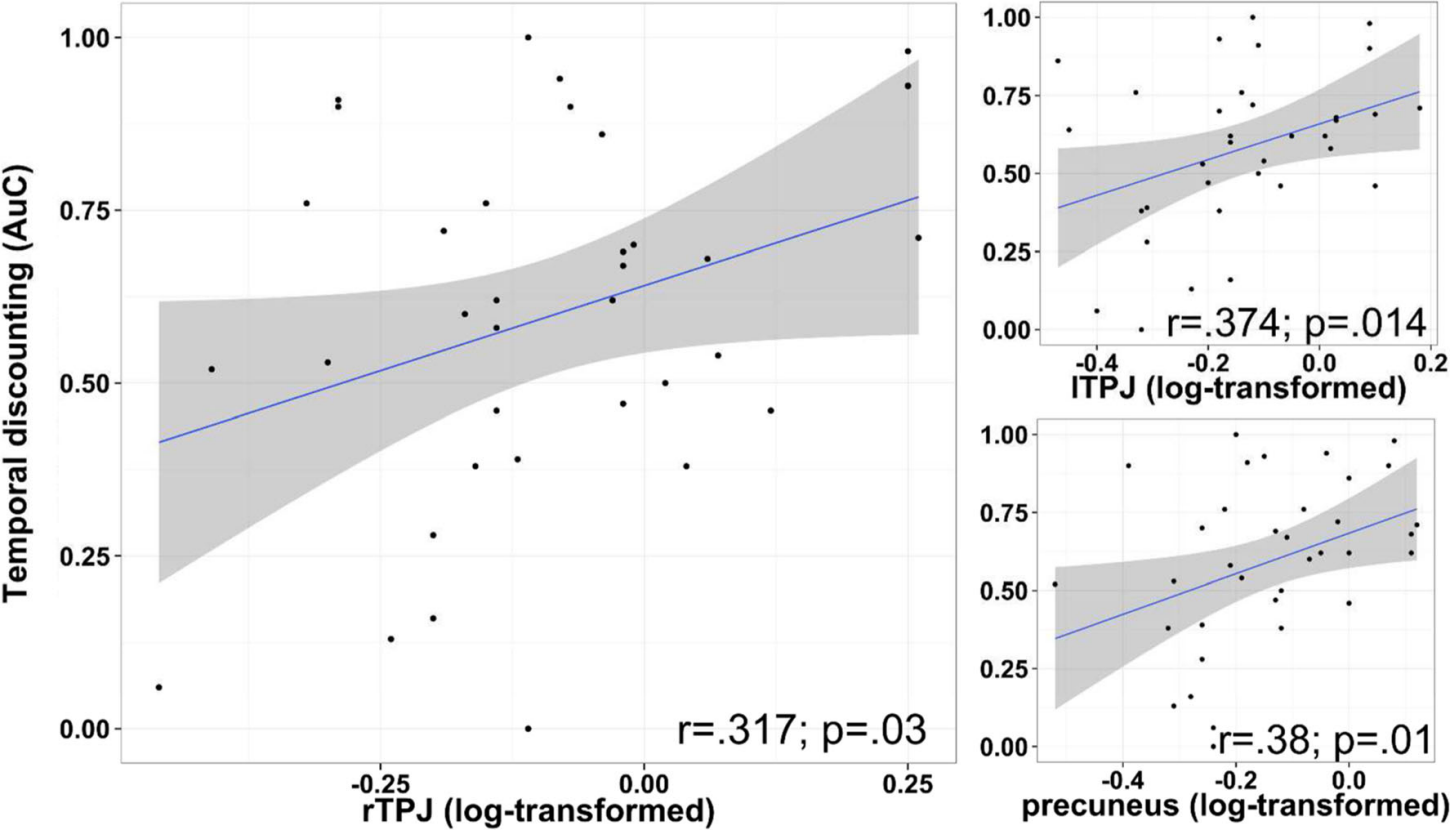
Scatterplots of temporal discounting AuC rates and averaged percentage signal change of False Belief > FACT in localized clusters

A GLM was run using condition regressors of “IMM” and “DEL” containing the first 32 trials of each, and the rest in “Excluded,” convolved with the canonical HRF. ROI analysis was conducted in MNI standard space to facilitate PPI analysis combining localized rTPJ cluster masks with standard space masks. ROI analysis was applied to two masks: localized rTPJ cluster masks (registered to MNI space using FEAT), and a 12-mm sphere in the vmPFC (see Fig. 2b). To focus on vmPFC processes related to representations of future rewards (i.e., those relevant to the SMIP), a meta-analysis was conducted using the MKDA method (Wager, Lindquist, Nichols, Kober, & Van Snellenberg, 2009) on coordinates reported in six studies using the same paradigm to cue imagination of delayed rewards during intertemporal choices (identified peak: [−4, 52, −10]; Fig. S1 and Table S1 in Supplementary Materials). The mean contrast values of DEL and IMM conditions (minus implicit baseline) in these ROIs were extracted with MarsBaR.

Generalized psychophysiological interaction (gPPI; McLaren, Ries, Xu, & Johnson, 2012) analysis was conducted with the gPPI toolbox in SPM8 to examine functional connectivity between the rTPJ and vmPFC. For each subject, we deconvolved the time series of the first eigenvariate of the blood-oxygen-level-dependent (BOLD) signal in the rTPJ seed region in standard space. The PPI regressor of each condition was calculated as the product of the seed region estimated neural response and the condition. We then performed GLM analysis including the condition and their respective PPI regressors, as well as the estimated neural response regressor of the seed region. The contrast images between the PPI regressor of the IMM and DEL condition from each participant were taken to a second-level one-sample *t* test on estimates of condition-dependent coupling between spatially averaged activity in the rTPJ seed region and voxels in the vmPFC sphere mask (small volume correction [SVC], FWE-corrected *p* < .05).

## Results

### Data cleaning and sample selection

Localizer task: participants’ whose false-belief regions could not be localized were excluded from analysis involving that region (final sample size: 36 for rTPJ and precuneus, 35 for lTPJ). Estimates of rTPJ_FB_ response were log-transformed to reduce the positive skew observed in these data (Kolmogorov–Smirnov test: *p* = .016 to *p* > .2 after log transformation; estimates from the lTPJ and precuneus were also log-transformed).

Temporal discounting task (scanner): DEL versus IMM estimates from the rTPJ cluster ROI were normally distributed (Kolmogorov–Smirnov test, *p* = .11), but two participants’ estimates were identified as outliers (Tukey’s criteria of 2 × IQR) and hence excluded from analysis. Tests of directional hypotheses (does not include the PPI) are reported with one-tailed p-values.

### fMRI: False-belief localization results

**Fig. 3:**
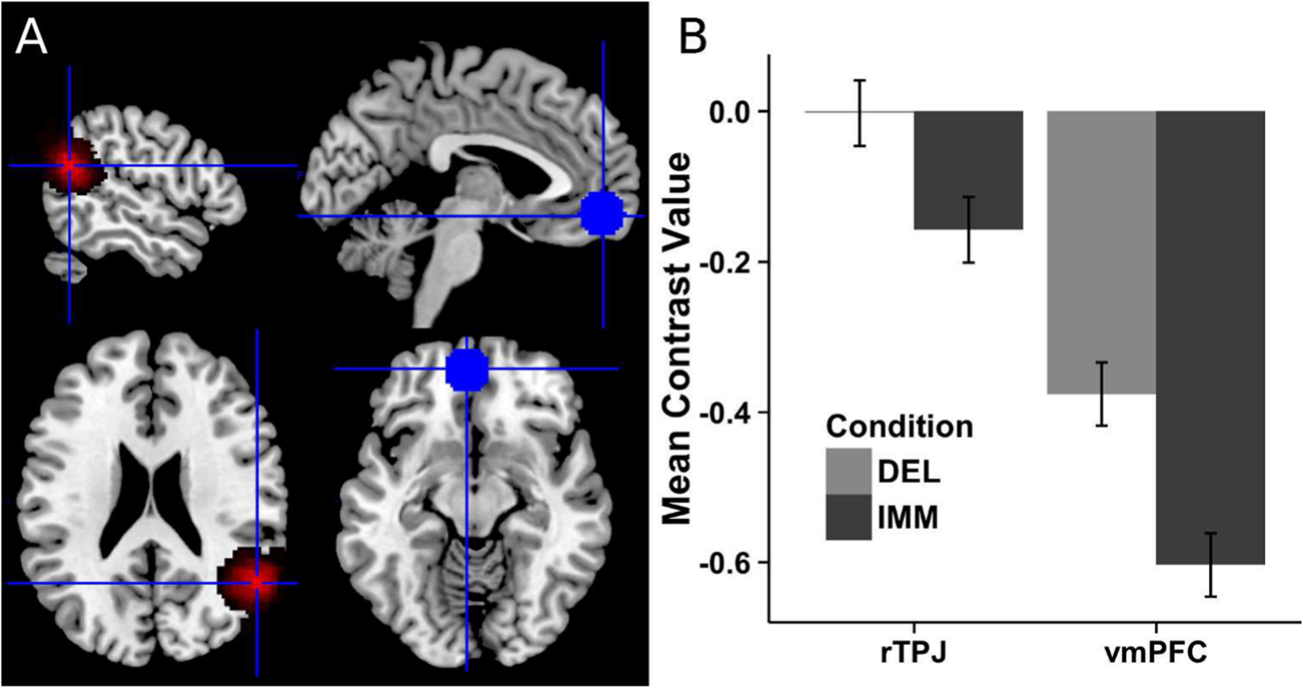
**a** Individuals’ rTPJ cluster masks overlaid in standard space (left) and the meta-analytically derived vmPFC sphere mask (right). **b** Activation differences between choice conditions (error bars: within-subject *SE*). (Color figure online)

Localized rTPJ cluster masks had peak overlap in MNI space at [52, −56, 18] (see Fig. 3a), close to the coordinate reported in a meta-analysis of false-belief tasks [50, −53, 21] (Decety & Lamm, 2007). In these clusters, the extracted rTPJ_FB_ signal had a mean value of .866%, *SE* = .07%. The correlation between rTPJ_FB_ and accuracy in false-belief trials (*M* = 62%, *SE* = .03) was at trend level, *r* = .225, *p* = .099, CI [−.11, .52]. No significant correlation was found between false-belief accuracy and temporal discounting rates, *r* = −.125, *p* = .234, CI [−.44, .21]. See Supplementary Materials for whole-brain analysis results.

### Correlation between rTPJ_FB_ and temporal discounting

A significant correlation was found between the outside scanner temporal discounting task AuC (*M* = .59, *SE* = .043) and rTPJ_FB_, *n* = 36, *r* = .32, *p* = .0288, CI [−.01, .59] (see Fig. 2) (lTPJ_FB_, *n* = 35, *r* = .37, *p* = .014, CI [.04, .63]; *n* = 36, precuneus_FB_, *r* = .392, *p* = .009, CI [.07, .64]), which remained significant in the temporal discounting scanning session subsample, *n* = 23, *r* = .375, *p* = .032, CI [−.04, .68]. Note that correlations with temporal discounting rates from the scanner task were not conducted, as immediate reward options in this version of the task were predetermined to facilitate data collection and were not estimated using the double-limits algorithm, as needed to calculate new indifference points and temporal discounting rates. Bootstrapping permutations (1,000) were used to test if the correlation with rTPJ_FB_ was robust to violations of parametric assumptions (e.g., nonnormal data, small sample sizes), *r* = .317, *p* = .03, CI [−.01, .58]. The correlation was also significant with the commonly used log-transformed *k* parameter of the steepness of fitted hyperbolic temporal discounting curves (see Supplementary Materials), *r* = −.315, *p* = .031, CI [−.02, .58].

### fMRI temporal discounting results

In the ROI analysis, a significant difference was found in the direction of DEL > IMM in the rTPJ, *n* = 25, *t* = 2.064, *p* = .025, and vmPFC, *n* = 26, *t* = 2.69, *p* = .006 (see Fig. 3b); not significant in lTPJ or precuneus. The rTPJ-seeded PPI analysis indicate a significant effect of choice condition in the vmPFC in the direction IMM > DEL at peak [−12, 51, −15], *n* = 25, *t* = 4.49, SVC voxel-level *p*_FWE_ = .017. For thoroughness of reporting, an additional PPI analysis was conducted between the rTPJ and left dorsolateral prefrontal cortex, a region reportedly involved in self-control during intertemporal choices (Hare, Hakimi, & Rangel, 2014). See Supplementary Materials for further information and whole-brain analysis results.

## Discussion

In this article, we tested hypotheses of the SMIP framework, which outline how perspective-taking abilities relate to preferences in intertemporal choices. The framework proposes that the subjective value of delayed rewards relies on the efficacy of taking the perspective of the recipients of those rewards, i.e. future selves. Similar to social forms of perspective taking, it requires the ability to control egocentric bias. We found three pieces of evidence in support of this view. First, people exhibiting a higher rTPJ response during false-belief judgments, a putative neural marker of egocentric bias control, demonstrated less steep temporal discounting. Second, activation in the same false-belief localized rTPJ region was higher when delayed rewards were preferred over immediate rewards in the temporal discounting task. Third, functional connectivity between the rTPJ and vmPFC differed between immediate and delayed choices, broadly supporting the SMIP’s proposed mechanism that intertemporal preferences are formed by interactions between egocentric bias control and representations of future reward value.

These results complement those of Soutschek et al. (2016), extending the forms of egocentric bias control related to temporal discounting from visual perspective taking to neural markers of false-belief inferences in the current study. These results also extend the set of overlapping neural bases between perspective taking and temporal discounting, with lTPJ and precuneus activity during perspective taking also positively related to preferences for delayed rewards.

Evidently, the above interpretation of these data relies on the reverse inference of ascribing the rTPJ a function of egocentric bias control. While the use of reverse inference is not fallacious per se (Hutzler, 2014), it should be considered carefully. Neural markers of perspective taking have the advantage of being fully continuous and therefore more resistant to ceiling effects often seen in behavioral measures. Increased rTPJ activity has been shown to be associated with higher false-belief accuracy (Gweon et al., 2012; Kana et al., 2009). This trend was also observed in our data, providing some validation for rTPJ activity as a marker of egocentric bias control. Indeed, studies of children, who do not show the typical ceiling effects in false-belief accuracy, have shown positive links between this accuracy and preferences for delayed rewards (Launay et al., 2015; Marchetti, Castelli, Sanvito, & Massaro, 2014).

In line with previous findings, evidence for the SMIP claim that the vmPFC is involved in representing the subjective value of future rewards was found in the current task. Higher vmPFC activity was observed during delayed relative to immediate reward choices. In addition, the SMIP’s proposed mechanism for how egocentric bias control interacts with these future reward value representations to guide intertemporal preferences was supported by the finding that functional connectivity between the rTPJ and vmPFC was choice dependent. Connectivity between these regions has been reported before in terms of both white-matter tracts and functionally during resting state (Bzdok et al., 2012; Mars et al., 2012). One explanation for the present observation of higher functional connectivity between these regions when immediate rewards were preferred over delayed is that in the vast majority of these trials, the amounts of immediate options were higher than they were when delayed rewards were chosen. We speculate that this increased subjective value made the immediate rewards more tempting, and triggered compensatory efforts to exert self-control to choose delayed rewards by imagining their anticipated enjoyment/benefit via rTPJ-vmPFC coupling.

Besides egocentric bias control, the rTPJ has a suggested role in orienting attention toward stimuli relevant to task goals (Corbetta & Shulman, 2002; Serences et al., 2005). This apparent dual function of the rTPJ has spurred suggestions that both tasks share a form of attention reorienting (e.g., in orienting away from instantly accessible and distracting self-related information toward other-related information during perspective taking; Mitchell, 2008; Schuwerk, Schurz, Müller, Rupprecht, & Sommer, 2016). A similar attention reorienting function could also be at work during intertemporal choices. In any form of choice, it could be argued that a goal to maximize reward outcomes, irrespective of the time of receipt, becomes activated. In intertemporal choices, delayed rewards are larger and hence most relevant to this goal, with immediate rewards constituting distractions from this goal. This view of rTPJ function in orienting attention is therefore consistent with our finding of it being more activated when choosing delayed over immediate rewards.

The effects reported in this study provide support for a theoretically proposed overlap between neural processes involved in egocentric bias control during perspective taking and those promoting delayed reward preferences in intertemporal choices. One open question is whether this overlap generalizes from hypothetical rewards (as used in the current study) to real-world rewards, as reports have indicated both similarities (Johnson & Bickel, 2002; Madden et al., 2004) and differences in these choice types (Matusiewicz, Carter, Landes, & Yi, 2013). Future studies should examine this question, and further test the generalizability of these results in larger samples, using different measures of egocentric bias control, and extend them to psychopathological groups marked by relevant deficits in perspective taking or impulsive decision-making (e.g., autism, addiction).

## Electronic supplementary material

ESM 1 (PDF 5468 kb)
