# Supplementary material for: Thinking about others and the future: Neural correlates of perspective taking relate to preferences for delayed rewards
Source: Cogn Affect Behav Neurosci. 2017 Nov 13;18(1):35–42. doi: 10.3758/s13415-017-0550-8 (PMC5823963; doi:10.3758/s13415-017-0550-8)
Supplement: Supplementary file 1 — (PDF 5468 kb) [file 13415_2017_550_MOESM1_ESM.pdf]

SUPPLEMENTARY MATERIALS

Double limits algorithm

The double-limits algorithm is used to find indifference points to map individual temporal discounting curves (Johnson & Bickel, 2002). Indifference points are the amount at which immediate rewards and delayed rewards are subjectively equivalent to the participant. In this procedure, value boundaries are adjusted after each choice. At the start, two "top" boundaries of each delay are set at the maximum possible immediate reward option amount (GBP £100), and the two "bottom" at the minimum (£0), and these are adjusted to guide estimations of the amount of immediate rewards that is at the indifference point. After each choice the boundaries are adjusted, decreasing from the top following immediate option choices or increasing from the bottom following delay option choices. The boundaries therefore "narrow in" on the range of possible immediate reward amounts in which the indifference point is likely to lie at each delay. One of each of the top and bottom boundaries are adjusted first ("inner"), then the other ("outer") follows one choice behind if both choices are consistent, e.g. if a £90 immediate reward was chosen, then an immediate reward above this choice would have been chosen in the previous choice. This step ensures that participants' choices are reliable. If inconsistent consecutive choices are made, then the inner boundaries are reset back to the position of the outer boundaries, and these outer boundaries reset to starting positions. The immediate reward amount is chosen randomly from between outer boundary ranges. The indifference point for a given delay is reached when the two outer boundaries are within £2 of each other (indifference points estimated as the midpoint in this range), after which options for that delay are no longer presented.

Area-under-curve

Area-under-curves were calculated by submitting indifference-points to the "trapz" function of the "flux" package in R-Project. This calculates the area of trapezoids under consecutive indifference points, beginning with a point of £100, i.e. the undiscounted delayed reward value.

Hyperbolic model fitting

Temporal discounting *k* rates were estimated by fitting a hyperbolic model [$v = V / (1 + kD)$, v = discounted delayed amount, V = undiscounted delayed amount, D = delay] to each participant's indifference-points using the "nls" non-linear model-fitting package in R-Project (*k* starting value = .001). As is standard practise (Myerson & Green, 2001), the distribution of these *k* values was

skewed, and were therefore log-transformed for parametric analysis.

Temporal discounting scanner task

Inside the scanner, immediate options were presented in the value ranges £5-15 above and below the indifference-points from the outside scanner data. Five equally spaced values along these ranges were presented as immediate reward options, and repeated until 32 trials (balanced across delays) were collected in which each of immediate and delayed rewards were chosen. If participants' indifference-points changed inside the scanner (e.g. delayed option selected when, based on outside scanner data, an immediate option was expected), indifference-points were automatically readjusted accordingly, and value ranges of subsequent immediate options recalculated, thereby ensuring the minimum 32 trials were collected in each condition. The task was run on PsychToolbox on MATLAB 2012b.

Multilevel Kernel Density Analysis (MKDA) meta-analysis

The MKDA meta-analysis algorithm (http://wagerlab.colorado.edu/tools) identifies spatial overlap of coordinates by placing 10 mm spheres around them and calculating the density of overlapping spheres (Kober et al., 2008). Monte Carlo simulations (1000 iterations) were used to control for family-wise error (FWE). Significantly overlapping coordinates were identified as density exceeding a voxel-wise height threshold of $p < .05$ of the resulting Monte Carlo simulated maps.

The MKDA meta-analysis method was used to identify the vmPFC region specifically related to representations of future rewards during intertemporal choices. This behavior is formally targeted by a paradigm developed by Peters and Büchel (2010), and has been used in five further studies (Benoit, Gilbert, & Burgess, 2011; Hu, Kleinschmidt, et al., 2017; Hu, Uhle, et al., 2017; Sasse, Peters, & Brassen, 2017; Sasse, Peters, Büchel, & Brassen, 2015). Coordinates of cluster peaks in the vmPFC region (BA10/BA11) showing increased activation in the contrast [Episodic future thinking > Baseline] were submitted to the meta-analysis (see Table S1 for coordinate list).

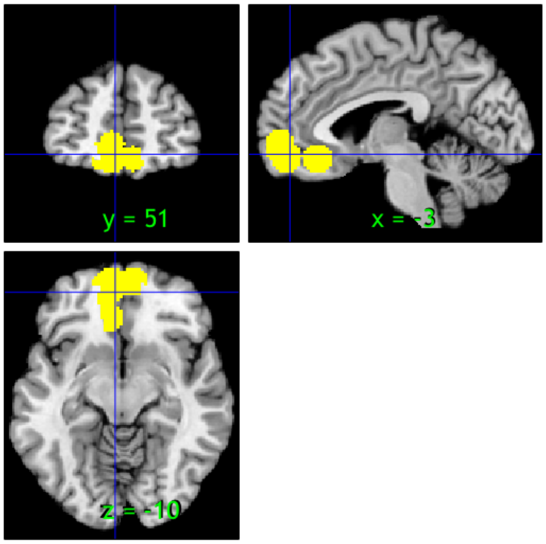

Figure S1. Spheres centered at the six vmPFC coordinates submitted to meta-analysis (crosshairs at peak overlap coordinate).

Table S1. Coordinates submitted to MKDA meta-analysis

MNI x,y,z	side	contrast	study
-6 58 -6	L	Episodic > Control	Sasse et al. (2015)
-10 52 -10	L	Episodic > Control (both groups)	Sasse et al. (2017)
8 57 -12	R	Episodic > Control (both groups)	Hu et al. (2016)
-5 53 -12	L	Episodic > Control (both groups)	Hu et al. (2017)
-6, 57, -3	L	Episodic > Control	Benoit et al. (2011)
-8 34 -12	L	Episodic > Control	Peters and Büchel (2010)

fMRI: whole-brain false belief analysis

To check main effects of the False Belief task, functional images were normalized to MNI space, and smoothed at 5 mm FWHM, and using FEAT, clusters from the False Belief > FACT contrast were identified at height threshold $z = 2.3$, cluster threshold $p < .05$, Gaussian Random Field FWER corrected. Whole-brain analysis identified clusters from the contrast of False Belief > FACT trials in the rTPJ [52, -66, 20] (voxels = 831, $z = 3.69$, $p < 0.01$), lTPJ [-64, -58, 14] (voxels = 390, $z = 4.34$, $p = 0.03$), precuneus [6, -66, 14] (voxels = 2147, $z = 8.64$, $p < 0.01$), and dorsomedial prefrontal cortex [30, 28, 56] (voxels = 2768, $z = 10.6$, $p < 0.01$) (Figure S2).

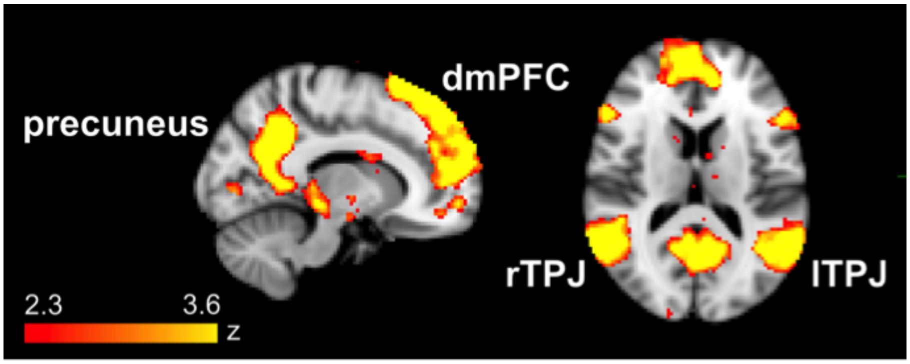

Figure S2. Whole-brain ToM tasks results from False Belief > FACT trials.

fMRI: whole-brain temporal discounting analysis

Whole-brain analysis was conducted at uncorrected cluster-forming threshold of $p < 0.005$, and corrected cluster-level FDR threshold of $p < .05$. Whole-brain analysis identified a cluster from the contrast of DEL > IMM trials with peaks in the left dorsal anterior cingulate gyrus [-9, 27, 18], medial prefrontal cortex [0, 63, 6], and pregenual cingulate gyrus [-6, 45, 9] (Figure S3).

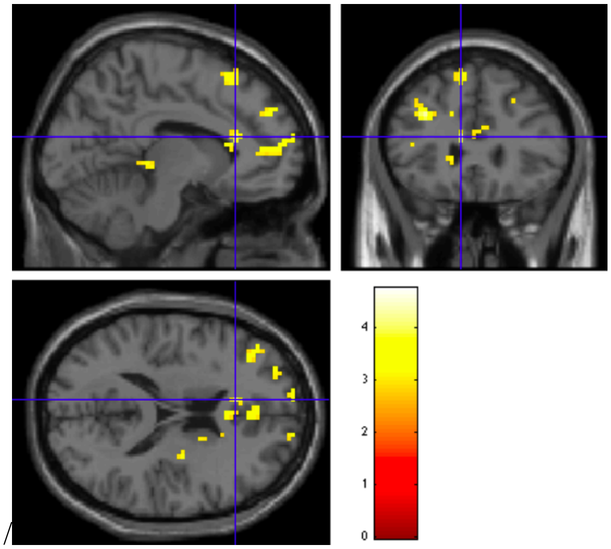

Figure S3. Whole-brain temporal discounting results of the contrast DEL > IMM (color bar indicates *t* statistic).

Whole brain parametric analysis was conducted to identify clusters of activity positively related to signals of net subjective value across all trials. Net subjective value refers to the subjective value of chosen reward options minus unchosen reward options, and subjective immediate reward value refers to the objective value of the immediate reward, and subjective delayed reward value refers to the indifference-point at that delay. See Table S2 and Figure S4 for

results. The whole brain parametric analysis regressing the subjective value of delayed rewards only (i.e. indifference-points) across all trials yielded no significant results.

Table S2. fMRI results of the temporal discounting parametric analysis of net subjective value.

cluster		peak			MNI				
pFDR	voxels	pFDR	T	Z	x,y,z	hemi	region	BA	Fig
0.005	146	0.172	6.14	4.68	-45 21 36	L	medial frontal gyrus	9	1
		0.714	3.5	3.1	-57 12 33		inferior frontal gyrus	9	
		0.881	3.2	2.88	-54 24 24			45	
0.008	125	0.498	5.18	4.18	-30 -63 54	L	superior parietal lobule	7	2
		0.881	3.2	2.88	-39 -66 48			7	
		0.885	3.18	2.86	-36 -54 39		inferior parietal lobule	40	
0.015	103	0.498	5.01	4.08	3 -30 33	R	posterior cingulate cortex	23	3
		0.689	3.81	3.32	0 -36 21			23	
0.002	192	0.539	4.76	3.93	33 -57 48	R	superior parietal lobule	7	4
		0.67	4.25	3.62	39 -66 42		inferior parietal lobule	7	
		0.689	3.86	3.36	30 -72 39		precuneus	19	
0	273	0.633	4.52	3.78	15 33 51	R	dorsomedial prefrontal cortex	8	5
		0.67	4.28	3.63	0 27 45			8	
		0.67	4.12	3.53	-3 36 42	L	supplementary motor area	6	

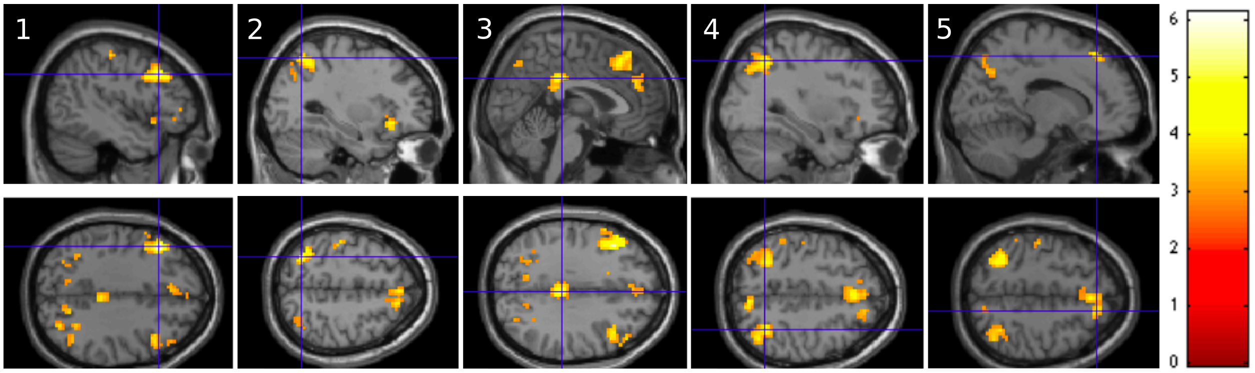

Figure S4. Parametric analysis of net subjective value in the temporal discounting task (see Table S2 for details on clusters referenced by panel numbers; color bar indicates *t* statistic).

fMRI: PPI temporal discounting analysis

An additional PPI analysis was conducted to examine functional connectivity between the rTPJ and left dorsolateral prefrontal cortex (lDLPFC). The lDLPFC is a region generally associated with abilities of self-control when impulsive decision-making is a risk, such as inhibiting cigarette cravings (Hayashi, Ko, Strafella, & Dagher, 2013), prepotent motor responses (Steinbeis, Bernhardt, & Singer, 2012), and immediate monetary reward options (Figner et al., 2010; Hare, Hakimi, & Rangel, 2014; McClure, Ericson, Laibson, Loewenstein, & Cohen, 2007). To investigate the possibility that activation of the rTPJ region, claimed here to be involved in egocentric bias control, underpins preferences for delayed rewards by enhancing self-control processes, we examined how functional connectivity between the rTPJ and the lDLPFC changed across choice conditions.

To spatially define the lDLPFC region, a 12 mm sphere centered on coordinates reported to be involved in self-control during intertemporal choices was chosen [x = -36, y = 30, z = 27] (Hare, Hakimi, & Rangel, 2014). Using this sphere as a SVC, a trend-level difference in functional connectivity was found between individuals' rTPJ cluster ROIs (localized in the false belief task) and a peak in the lDLPFC sphere in the direction of DEL > IMM [x = -33, y = 36, z = 21], n = 25, $t = 3.64$, SVC voxel-level $p_{\mathrm{FWE}} = .088$.

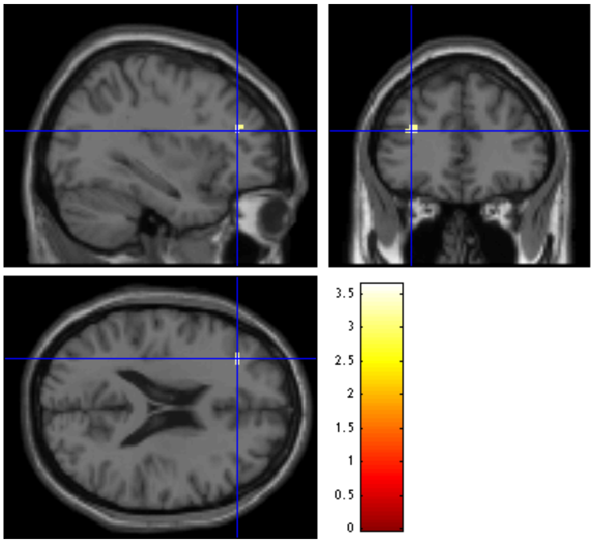

Figure S5. PPI analysis result of increased functional connectivity between the rTPJ seed region and lDLPFC for DEL > IMM.

REFERENCES

Benoit, R. G., Gilbert, S. J., & Burgess, P. W. (2011). A neural mechanism mediating the impact of episodic prospection on farsighted decisions. The Journal of Neuroscience: The Official Journal of the Society for Neuroscience, 31(18), 6771–6779. https://doi.org/10.1523/JNEUROSCI.6559-10.2011

Figner, B., Knoch, D., Johnson, E. J., Krosch, A. R., Lisanby, S. H., Fehr, E., & Weber, E. U. (2010). Lateral prefrontal cortex and self-control in intertemporal choice. Nature Neuroscience, 13(5), 538–539. https://doi.org/10.1038/nn.2516

Hare, T. A., Hakimi, S., & Rangel, A. (2014). Activity in dlPFC and its effective connectivity to vmPFC are associated with temporal discounting. Frontiers in Neuroscience, 8. https://doi.org/10.3389/fnins.2014.00050

Hayashi, T., Ko, J. H., Strafella, A. P., & Dagher, A. (2013). Dorsolateral prefrontal and orbitofrontal cortex interactions during self-control of cigarette craving. Proceedings of the National Academy of Sciences of the United States of America, 110(11), 4422–4427. https://doi.org/10.1073/pnas.1212185110

Hu, X., Kleinschmidt, H., Martin, J. A., Han, Y., Thelen, M., Meiberth, D., … Weber, B. (2017). A Reduction in Delay Discounting by Using Episodic Future Imagination and the Association with Episodic Memory Capacity. Frontiers in Human Neuroscience, 10. https://doi.org/10.3389/fnhum.2016.00663

Hu, X., Uhle, F., Fliessbach, K., Wagner, M., Han, Y., Weber, B., & Jessen, F. (2017). Reduced future-oriented decision making in individuals with subjective cognitive decline: A functional MRI study. Alzheimer's & Dementia: Diagnosis, Assessment & Disease Monitoring, 6, 222–231. https://doi.org/10.1016/j.dadm.2017.02.005

Johnson, M. W., & Bickel, W. K. (2002). Within-Subject Comparison of Real and Hypothetical Money Rewards in Delay Discounting. Journal of the Experimental Analysis of Behavior, 77(2), 129–146. https://doi.org/10.1901/jeab.2002.77-129

Kober, H., Barrett, L. F., Joseph, J., Bliss-Moreau, E., Lindquist, K., & Wager, T. D. (2008). Functional grouping and cortical–subcortical interactions in emotion: A meta-analysis of neuroimaging studies. NeuroImage, 42(2), 998–1031. https://doi.org/10.1016/j.neuroimage.2008.03.059

McClure, S. M., Ericson, K. M., Laibson, D. I., Loewenstein, G., & Cohen, J. D. (2007). Time Discounting for Primary Rewards. Journal of Neuroscience, 27(21), 5796–5804. https://doi.org/10.1523/JNEUROSCI.4246-06.2007

Peters, J., & Büchel, C. (2010). Episodic future thinking reduces reward delay discounting through an enhancement of prefrontal-mediotemporal interactions. Neuron, 66(1), 138–148. https://doi.org/10.1016/j.neuron.2010.03.026

Sasse, L. K., Peters, J., & Brassen, S. (2017). Cognitive Control Modulates Effects of Episodic Simulation on Delay Discounting in Aging. Frontiers in Aging Neuroscience, 9. https://doi.org/10.3389/fnagi.2017.00058

Sasse, L. K., Peters, J., Büchel, C., & Brassen, S. (2015). Effects of prospective thinking on intertemporal choice: The role of familiarity: Future Event Construction and Delay Discounting. Human Brain Mapping, 36(10), 4210–4221. https://doi.org/10.1002/hbm.22912

Steinbeis, N., Bernhardt, B. C., & Singer, T. (2012). Impulse Control and Underlying Functions of the Left DLPFC Mediate Age-Related and Age-Independent Individual Differences in Strategic Social Behavior. Neuron, 73(5), 1040–1051. https://doi.org/10.1016/j.neuron.2011.12.027
